# Retrospective comparison of the effects of laryngeal mask and endotracheal tube on some cardio-respiratory variables in pet rabbits undergoing anaesthesia for elective gonadectomy

**DOI:** 10.1186/s13028-023-00673-2

**Published:** 2023-03-01

**Authors:** Luca Bellini, Magdalena Schrank, Irene Alessandra Veladiano, Barbara Contiero, Antonio Mollo

**Affiliations:** Department of Animal Medicine, Production and Health, University of Padova, Viale dell’Università 16, 35020 Legnaro, PD Italy

**Keywords:** Buprenorphine, Endotracheal tube, Laryngeal mask, Gonadectomy, Rabbits

## Abstract

**Background:**

Endotracheal intubation in rabbits is challenging and supraglottic airway devices, such as laryngeal masks (LMA), represent an alternative as they are easy to insert, and do not stimulate the larynx requiring therefore a lighter plane of anaesthesia for their insertion and positioning than the endotracheal tubes. We investigated whether, compared to an endotracheal tube, the LMA can reduce the negative effects of general anaesthetics on some cardiovascular and respiratory parameters routinely monitored in rabbits anaesthetized for elective gonadectomy. The records of 21 adult mixed breed pet rabbits were collected retrospectively. Rabbits were divided in two groups based on the type of airway device used. A laryngeal mask secured the airway in group LMA (n = 11), and in group ETT (n = 10) an endotracheal tube was used. The amount of propofol used before successful insertion of the airway device was recorded. A pitot-based spirometer was connected and ventilatory variables were measured immediately after insertion. Pulse rate, non-invasive arterial blood pressure, haemoglobin oxygen saturation, respiratory rate, end-tidal carbon dioxide and volatile anaesthetic consumption were also monitored during the surgical procedure; extubation time was noted as well.

**Results:**

The use of LMA required significantly less propofol (0.8 to 4 mg/kg) for insertion than the ETT (1.2 to 5.6 mg/kg), and the difference was statistically significant (P < 0.01). No differences were observed in ventilatory variables measured immediately after airway positioning. Intraoperatively, there were no differences between groups for respiratory and cardiovascular variables, and amount of isoflurane administered. In all rabbits mean and diastolic blood pressure progressively decreased during surgery. Mean extubation time was shorter in group LMA (6 ± 2 min) than group ETT (8 ± 3 min, P < 0.01).

**Conclusion:**

The airway device did not clinically affect the cardiovascular and respiratory variables during anaesthesia. Intraoperative hypoventilation was observed in most rabbits regardless of the device being used; therefore ventilatory support may be required. Rabbits with the laryngeal masks were extubated earlier.

## Background

The most common surgical procedure performed in pet rabbits is female and male neutering [1]. These animals are frequently sedated intramuscularly for venous catheterization or before inhalational anaesthesia as stress and anxiety may predispose to intraoperative apnoea and cardiac instability [2, 3]. Frequently injectable anaesthetics are combined with alpha 2 agonists for sedation in rabbits, but this combination may lead to respiratory impairment and poor arterial oxygen content [1, 4, 5].

Previously we reported a sedation protocol to chemically immobilize pet rabbits for non-painful procedures [6]. Dexmedetomidine (50 µg/kg) and midazolam (0.6 mg/kg) resulted in adequate restraint in all rabbits for abdominal ultrasound, with minimal depression of pulse rate and Doppler arterial pressure; moreover, none of the animals had a haemoglobin oxygen saturation (SpO_2_) below 94% despite breathing room air.

In recent years, awareness towards pain relief during surgical procedures in lagomorphs has increased among veterinary practitioners [7]. Buprenorphine is an analgesic commonly administered during anaesthesia in rabbits and it has a long duration of action with a negligible effect on the heart rate [3, 4, 7]. In this species, buprenorphine decreases respiratory rate and impairs ventilation, especially with the concurrent administration of sedatives and volatile anaesthetics [3, 4, 8]. This may lead to clinically relevant hypoxia, and a source of oxygen and ventilatory support should be available [4]. In rabbits, achieving a safe control of airways to provide oxygen or to ventilate is challenging [9]. Endotracheal intubation requires training and regular practice; therefore the use of alternative airway devices, such as laryngeal masks (LMA), has been considered instead [10–14]. Most of those studies experimentally assess the insertion technique and performance of the LMA under mechanical ventilation. The intraoperative impact of those devices in spontaneously ventilating rabbits has received limited investigation. Moreover, rabbits are particularly sensitive to the cardiovascular depressant effects of general anaesthetics, particularly volatile anaesthetics, and their associated hypotension [15, 16]. In dogs, the insertion of the LMA required a lower dose of propofol compared to an endotracheal tube [17] and in young adult patients undergoing surgery, the LMA was tolerated at lower isoflurane concentration than those required with endotracheal intubation [18].

The aim of this study was to evaluate if the use of LMA or endotracheal tube (ETT) to secure the airway affected some cardiovascular and respiratory parameters commonly measured during volatile anaesthesia in pet rabbits undergoing routine gonadectomy. We hypothesized that the use of LMA could be associated with a reduced requirement of volatile agents, attenuating the dose-dependent cardiovascular and respiratory depression reported with isoflurane in rabbits [19].

## Methods

### Animals

The anaesthetic records of 21 adult (15 females and 6 males) mixed breed pet rabbits classified as American Society of Anesthesiologists (ASA) physical status 1, collected by the authors for two previous studies [15, 20], were retrospectively assessed. The animals were considered healthy based on clinical history and physical examination, and all rabbits did not show abnormal respiratory sounds or nasal and ocular discharge. Weight and age of the animals ranged from 2.5 to 4.6 kg and between 8 and 14 months, respectively. The rabbits were scheduled for elective neutering under general anaesthesia with isoflurane. All animals were admitted to the Clinical Teaching Hospital of the University of Padova in the morning and the surgery was performed in the afternoon. Food and water were available until premedication was administered.

### Study design

Rabbits were divided into two groups based on the device used to secure the airways with an online randomiser (Research Randomizer 4.0). Group ETT had airways secured with an uncuffed endotracheal tube, while in group LMA a laryngeal mask was used. The airway devices used were: in group ETT, a 3-3.5 internal diameter ETT (Ruschelit Rüsch; Teleflex, Limerick, PA, USA) and in group LMA, a size 1 laryngeal mask (Soft Seal Portex; Smiths Medical, Minneapolis, MN, USA). Both devices were inserted blindly by the same anaesthetist (LB), with the rabbit in sternal recumbency and the head being held extended over the neck. If the anaesthetist considered the tube adequately inserted, a mainstream capnometer was connected to the airway device and the intubation was considered successful if end-tidal carbon dioxide (PE'CO_2_) was measured. Absence of detection of PE'CO_2_ or removal of the airway device to try a new insertion were considered unsuccessful attempts. A maximum of three attempts were performed blindly before inserting the device under direct endoscopic visualization.

### Anaesthesia

Rabbits were premedicated intramuscularly with a standard protocol that included 50 µg/kg dexmedetomidine (Dexdomitor; Orion Corporation, Espoo, Finland) and 0.6 mg/kg midazolam (Midazolam-hameln; Hospira Italia, Naples, Italy). All drugs were mixed in the same syringe and injected in the lumbar muscles. As rabbits lost the righting reflex, the abdomen in females and the inguinal and scrotal region in males were clipped; a 26-gauge (Delta Ven 1, Delta Med Spa, Viadana, Italy) over the needle catheter was inserted aseptically in the cephalic vein and buprenorphine (Buprenodale; Dechra Ltd., Skipton, UK) at the dose of 0.05 mg/kg was administered intravenously (IV). The effect of premedication was scored according to a previously described scale [6]. The descriptors of the scale were: posture from 0 (normal) to 5 (lying dorsally, not moving when stimulated); resistance to being rolled in dorsal recumbency from 0 (strong/normal resistance) to 3 (no resistance ); jaw muscle tone 0 (normal) or 1 (no resistance to mouth opened); palpebral reflex from 0 (normal) to 2 (absent). Oxygen at 1 L/min was administered via a Mapleson E breathing system placed close to the nose for at least 5 min and after, larynx was blindly sprayed with 0.2 mL of lidocaine (Lidocaina 2%; Ecuphar Italia S.r.l., Milan, Italy, diluted to 5 mg/mL) before the first attempt of insertion of the device. If the score was equal or higher than 9 out of 11, insertion of the LMA or the endotracheal tube was attempted, otherwise propofol (Vetofol; Norbrook Laboratories Ltd., Newry, Northern Ireland) was administered IV manually at 0.2 mg/kg every 10 seconds, until the score was considered adequate.

As the airways were secured, a pitot base spirometer connector (Pedi-Lite Spirometry Sensor; GE Healthcare, Helsinki, Finland) was connected to measure the tidal volume (Vt). After, the rabbits were placed in dorsal recumbency and connected to a circle breathing system with paediatric tubes; anaesthesia was maintained with isoflurane (Isoflo; Abbott Laboratories Ltd., Maidenhead, UK) carried in a mixture of oxygen and air (FiO_2_ = 0.5); the surgical area was aseptically prepared. Initial flow rate was 1 L/min which was decreased to 0.5 L/min. End-tidal concentration of isoflurane was adjusted to maintain an adequate anaesthetic level minimizing the occurrence of surgery-induced cardiovascular and respiratory changes or spontaneous movement. Rabbits were maintained in spontaneous ventilation throughout anaesthesia, and manual ventilation at six breaths/min and a peak pressure of 10 cm H_2_O was provided if apnoea was present or the respiratory rate was lower than < 6 breaths/min. A portable pulse oximeter (EDAN VE-H100B; Edan USA, San Diego, CA, USA), with a clip probe placed over the ear, measured the pulse rate (PR) and SpO_2_, while the PEʹCO_2_, the end-tidal concentration of isoflurane (FEʹIso) and the respiratory rate (RR) were obtained from a multiparameter monitor (Datex S/5; GE Healthcare, Helsinki, Finland). Non-invasive systemic arterial blood pressure was measured by an oscillometric device (PetTrust Blood Pressure Monitor; BioCare, Taoyuan City, Taiwan). The cuff was placed on the forelimb proximal to the carpus; the correct size of the cuff was obtained by a ruler provided by the manufacturer. A mixture of lactated Ringer’s solution and 5% dextrose (50:50) was infused at 5 mL/kg/h during anaesthesia. Before the beginning of the surgery the rabbits received meloxicam (1 mg/kg; Metacam; Boehringer Ingelheim, Milan, Italy) and enrofloxacin (5 mg/kg; Baytril 25 mg/mL injection solution; Bayer Spa, Milan, Italy) subcutaneously.

### Measurements

Data collected from the anaesthetic records included: pulse rate, respiratory rate and rectal temperature that were recorded before premedication and the dose of propofol to safely secure the airways. After the insertion of the airway device, the pitot spirometer connector with a respiratory gas sampling port was attached to the multiparameter monitor for RR, PEʹCO_2_ and Vt measurements. After the breathing system was connected, RR, PE'CO_2_, FE'Iso, PR, systolic arterial pressure (SAP), mean arterial pressure (MAP) and diastolic arterial pressure (DAP) were recorded before incision (Tbefore), 5 min after skin incision (Tincision), during surgery (Tsurgery) and at the end of the surgery (Tend). All variables measured during surgery were recorded every 5 min. At the end of surgery, the animals were disconnected from the breathing system and atipamezole was injected at 0.5 mg/kg subcutaneously. Extubation time was considered as the time from disconnection of the anaesthesia machine to airway device removal, after active chewing or swallowing was noted.

### Statistical analysis

The sample size was calculated from a previous study evaluating the dose of propofol required to insert an LMA or an ETT in dogs [17]. Based on those data, a decrease of 40 ± 25% for the dose of propofol and isoflurane required to correctly place the airway device and maintain an adequate anaesthetic depth during surgery was considered clinically relevant. The analysis returned a minimum sample size of nine rabbits per group with a power and α set at 0.9 and 0.05, respectively (https://www.imim.es/ofertadeserveis/software-public/granmo/).

For the statistical analysis, variables measured before skin incision (Tbefore) and during surgery (Tsurgery) were averaged and used as raw data. Normally distributed data were expressed as a mean ± standard deviation, otherwise a median (min-max) was used. Differences between groups to loss of righting reflex, the total dose of propofol administered, basal and post induction RR, PE'CO_2_, Vt and rectal temperature, rectal temperature at recovery, and extubation time were evaluated with an analysis of covariance linear model with airway device, gender as fixed effects and weight as a covariate, or with a Wilcoxon non-parametric analysis of variance, as appropriate. A repeated linear mixed model was used to analyse PR, RR, PE'CO_2_, FEʹIso, SAP, MAP and DAP over time. Gender, airway device, time and their interaction were inserted as fixed effects while subject was included as random repeated effects. Bonferroni post hoc pairwise comparison test between least squares means was used if statistical differences were present.

Analyses were performed with a statistical package (SAS V.9.1; SAS Institute Inc, Cary, NC, USA) and P < 0.05 was considered statistically significant. The statistician (BC) was not involved in data collection.

## Results

Twenty-one anaesthetic records were revised and rabbits included in group ETT and LMA were 10 and 11, respectively.

No differences were observed in both groups regarding weight (P = 0.57) which was 3.4 ± 0.6 and 3.3 ± 0.7 kg for group ETT and LMA, respectively. No adverse effects were recorded during sedation, surgical procedure or in the postoperative period. The median time between the initial attempt to successfully insertion of the airway device was 125 (61–282) and 0 (0–200) seconds in group ET and group LMA, respectively. Anaesthesia time was 39 ± 8 and 40 ± 9 min (P = 0.89) in group ETT and group LMA, respectively. The laryngeal mask was considered adequately placed and not dislodged throughout the anaesthetic procedure despite changes in the animals position, as a normal capnograph trace was present.

Table 1 shows the variables evaluated between the groups after administration of sedatives and at the end of anaesthesia. No statistical differences were detected between groups except of a lower dose of propofol used (P < 0.01) and a shorter extubation time (P < 0.01) in group LMA compared to group ETT. Three animals in group LMA and 9 in group ETT received propofol to secure the airway. The score recorded in animals requiring propofol in group LMA was 3, 8 and 7. The nine animals in group ETT that had responded to tube insertion had a score above 10. Males had a statistically lower body temperature compared to females in recovery (37.3 ± 0.9 vs. 38.6 ± 0.8 °C; P < 0.01) but extubation time was unaffected by gender or weight. Haemoglobin oxygen saturation was continuously maintained over 95% from the loss of righting reflex to extubation.

**Table 1:** Results of the covariance linear model analysis (F-stat) or Wilcoxon non-parametric (W-stat) analysis of variance

			Airway device		Gender		Weight	
	ETT	LMA	F/W-stat	P-value	F-stat	P-value	F-stat	P-value
Loss of RiRef (sec)	391 ± 207	396 ± 145	0.02	0.89	0.00	0.97	0.84	0.37
Propofol (mg/kg)	2.3 (0-5.6)	0 (0-4)	8.82	< 0.01	–	–	–	–
RR (Breaths/min)	148 ± 43	136 ± 56	0.02	0.88	0.74	0.40	2.35	0.15
RR post induction (Breaths/min)	29 ± 7	37 ± 10	0.41	0.53	1.18	0.30	1.09	0.31
PE'CO2 (mmHg)	51 ± 8	41 ± 4	3.75	0.07	3.21	0.09	0.54	0.47
Vt (ml/kg)	6 (5-12)	7 (4-21)	0.16	0.69	–	–	–	–
Tinitial (°C)	39.1 ± 0.5	39.1 ± 0.4	1.72	0.21	4.28	0.06	0.00	0.99
Trecovery (°C)	38.3 ± 1.1	38.2 ± 0.9	1.44	0.25	12.76	< 0.01	1.82	1.82
Extubation time (min)	8 ± 3	6 ± 2	11.18	< 0.01	3.07	0.10	0.27	0.61

Data are reported as mean ± standard deviation or median (min-max). Respiratory rate was measured before and after the insertion of the airway device post anaesthesia induction; PE’CO_2_ and Vt were measured by a pitot spirometer connector with a respiratory gas sampling port attached to the airway device and connected to a multiparameter monitor

*ETT* endotracheal tube, *LMA* laryngeal mask airway device, *PE’CO*_2_ end-tidal CO_2_, *RiRef* righting reflex, *RR* respiratory rate, *Tinitial* temperature measured after the loss of righting reflex, *Trecovery* temperature measured at recovery, *Vt* tidal volume

The airway device used, the interaction between time and airway device and the gender did not affect PR, RR, PE'CO_2_, FEʹIso, SAP, MAP and DAP. Time had an effect on RR (P < 0.01), FE'Iso (P < 0.01), MAP (P = 0.02) and DAP (P = 0.01) (Tables 2 and 3). From baseline the RR decreased but remained unchanged throughout the anaesthesia (Table 2). At Tincision and Tsurgery the FEʹIso was higher than that measured at Tbefore in both groups; although not statistically significant, group LMA showed an anaesthetic consumption lower than group ETT (Table 2). No differences were detected for PR or SAP over the selected time points (Table 2). Both MAP and DAP showed a progressive decrease over time although no statistical differences were detected between groups (Tables 2 and 3).

**Table 2:** Respiratory and cardiovascular variables (mean ± standard deviation, median (min-max)) over time in rabbits under isoflurane anaesthesia

		Baseline	Before incision	At incision	During surgery	End of surgery
Respiratory rate (breath/minute)						
LMA		136 ± 56	31 ± 11	21 ± 8	26 ± 4	30 ± 14
ETT		148 ± 43	24 ± 8	17 ± 9	29 ± 5	40 ± 12
PE’CO_2_ (mmHg)						
LMA		–	45 ± 7	48 ± 14	49 ± 9	46 ± 8
ETT		–	52 ± 9	60 ± 15	54 ± 14	50 ± 15
FE’ISO (vol/vol%)						
LMA		–	0.2 (0–1)	0.5 (0-1.1)	0.7 (0.1–1.1)	0.2 (0-0.9)
ETT		–	0.1 (0-1.4)	0.8 (0.5–2.5)	0.9 (0.1–1.5)	0.7 (0.1–1.5)
Pulse rate (beats/minute)						
LMA		187 ± 29	190 ± 12	196 ± 13	201 ± 13	198 ± 9
ETT		178 ± 33	171 ± 27	186 ± 24	190 ± 25	191 ± 22
Systolic arterial pressure (mmHg)						
LMA		–	85 ± 14	92 ± 20	85 ± 16	91 ± 17
ETT		–	95 ± 21	102 ± 22	92 ± 19	91 ± 14
Diastolic arterial pressure (mmHg)						
LMA		–	53 ± 18	60 ± 17	48 ± 12	53 ± 17
ETT		–	63 ± 17	66 ± 15	54 ± 20	50 ± 17
Mean arterial pressure (mmHg)						
LMA		–	63 ± 14	71 ± 17	60 ± 14	65 ± 17
ETT		–	74 ± 17	76 ± 18	66 ± 19	63 ± 15

**Table 3:** Results of the linear repeated mixed model analysis for the cardiovascular and respiratory intraoperative variables

	Airway device		Time		Airway device x Time		Gender	
	F-stat	P-value	F-stat	P-value	F-stat	P-value	F-stat	P-value
Respiratory rate	0.03	0.87	49.04	< 0.01	0.10	0.98	0.56	0.47
PE*’*CO_2_	1.10	0.31	0.77	0.52	0.12	0.95	0.91	0.36
FE*’*ISO	1.38	0.26	6.86	< 0.01	1.63	0.20	0.03	0.87
Pulse rate	2.51	0.13	1.61	0.19	0.65	0.63	0.06	0.81
Systolic arterial pressure	2.03	0.17	2.16	0.11	2.31	0.09	0.07	0.79
Diastolic arterial pressure	0.63	0.44	3.97	0.01	1.65	0.19	0.600	0.45
Mean arterial pressure	1.26	0.28	3.55	0.02	2.08	0.12	1.26	0.28

*FE'ISO* fraction inspired of isoflurane, *PE'CO*_2_ end-tidal partial pressure of CO_2_

## Discussion

Sedation achieved with dexmedetomidine, midazolam and buprenorphine was considered adequate for laryngeal mask placement, with only few rabbits requiring additional propofol. The decision to use either a laryngeal mask or an endotracheal tube does not cause statistically relevant changes in the intraoperative cardiovascular and respiratory variables taken into consideration.

In our study, the animals included in the ETT group required more propofol than the one in the LMA group and similar results were reported in dogs [17]. In three rabbits of group LMA, airway device insertion required a variable dose of propofol, suggesting a lower effect of premedication in those subjects. The difference in the sensitivity to sedatives or anaesthetics in mixed-breed rabbits could explain this finding, as medetomidine and propofol showed a strain-dependent response in this species [21]. In this study, the induction technique might have influenced the final dose of propofol required to secure the airway. Low doses of propofol were repeated at 10 s intervals until the score was equal to or above nine before attempting intubation; prolonged induction time could have reduced anaesthesia depth as propofol has a short duration of action. Because propofol can cause apnoea, especially with a rapid administration or concurrent use of sedatives, induction was slow to avoid this occurrence.

In rabbits, propofol administration causes a decrease in respiratory rate [22]. Because more rabbits received boluses of propofol in group ETT, we expected a lower respiratory rate in these animals after induction, but there was no difference with group LMA. Plasma catecholamines level increases more after endotracheal intubation compared to supraglottic devices placement [23]; in our study, the sympathetic response to the endotracheal tube may have offset the respiratory depression due to propofol explaining the lack of statistical differences in the respiratory rate between groups. Moreover, regardless the device used, no differences were noted in tidal volume or the fraction expired of carbon dioxide after the insertion of the airway device. This suggests that the minute volume between groups was similar likely because the devices did not decrease the cross-sectional area of the larynx [13]. On the contrary, two studies detected an increase in the partial pressure of carbon dioxide in anaesthetized rabbits having the airway secured with a veterinary designed laryngeal mask for rabbits [13, 14]. In those studies, carbon dioxide rebreathing was suspected due to device leaking of expired gas or increase in dead space due to the LMA internal diameter. Compared to other supraglottic devices available in Veterinary Medicine, the laryngeal mask used in our study was designed for human paediatric patients. These devices may show less leaking during spontaneous ventilation and do not decrease the laryngeal cross-section area that remains similar to physiologic values for rabbits between 2.5 and 5 kg [12, 13]. Although not statistically significant, the end-tidal carbon dioxide in group ETT was higher. It could be due to the lower internal diameter of the endotracheal tube compared to the LMA, which may have led to an increase in the work of breathing with a mild hypoventilation [24].

Respiratory rate decreased in both groups after the loss of righting reflex as observed with alpha-2 sedation protocols in rabbits [6]. Buprenorphine at 0.03 mg/kg injected intravenously was associated with a decrease in the respiratory rate and hypoxaemia [8]. To avoid this occurrence, 100% oxygen was provided via a paediatric breathing system placed close to the nostrils as the rabbit lost the righting reflex.

The end-tidal concentration of isoflurane increased during surgery as observed in spay procedures in other species [25] although in our study the pulse and respiratory rate and the systemic arterial blood pressure remained constant throughout anaesthesia. This suggests that buprenorphine provided an adequate control of intraoperative nociceptive stimulation, minimizing the haemodynamic response to surgical manipulation [25]. After the airway device insertion, but before the beginning of surgery, the end-tidal concentration of isoflurane was similar between groups. Similarly, Iizuka et al. found that in dogs, the plasma concentration of propofol required to tolerate an endotracheal tube or a laryngeal mask was similar, and minimally affected by the airway device unless the application of an intense nociceptive stimulus led to a cardiovascular response [26]. Moreover, insertion of an airway device elicits a more intense haemodynamic response rather than its maintenance [27]. In the current study, dexmedetomidine improved the hypnosis, and it might have contributed to increasing the tolerance of the airway device, and minimising the haemodynamic response to surgery. Regardless of the differences in surgical procedure, the dose of isoflurane was similar between males and females at each time point. This suggests an adequate control of nociception by the dose of buprenorphine in both procedures preventing thereby increases in heart rate or systemic arterial blood pressure as observed in another study [25]. The end-tidal concentration of isoflurane, although not statistically significant different between groups, was lower in rabbits in which a laryngeal mask was used than in those that had an endotracheal tube. The lower concentration of isoflurane at the end of anaesthesia in the LMA group may have shortened the extubation time.

Most of the rabbits became hypercapnic during anaesthesia. Buprenorphine was reported to cause respiratory depression and hypoventilation that was enhanced by concurrent administration of sedatives [4, 8]. Moreover, rabbits are particularly sensitive to the ventilatory depressant effects of volatile agents and ventilatory support should be considered in such a condition [10]. Lack of differences between groups may reflect the depressive effect of sedatives and opioids administered.

Mean and diastolic systemic arterial blood pressure showed a reduction over time mainly during surgery and at the end of the procedure although no statistical differences were observed between groups. An increase in the concentration of volatile anaesthetic in that period may explain the results and confirms the high sensitivity of this species regarding the isoflurane-induced vasodilation [4, 15]. Regardless of the device used to secure the airways, values recorded for mean and systolic arterial blood pressures remained above 60 and 85 mmHg, respectively, in most rabbits. Although these values are considered clinically acceptable, in rabbits the oscillometric monitor used in this study showed to overestimate the real value of arterial blood pressure [15], suggesting that some rabbits could have been hypotensive. Indeed, 65 mmHg is the cut-off above which this oscillometric monitor identifies animals with an invasive mean arterial pressure > 60 mmHg [15]. The amount of isoflurane administered in our study was lower than that reported in rabbits receiving other alpha-2 agonist based protocols [28]. The sedation protocols allowed toleration of the airway device and it contributed to the decrease in anaesthetic requirement regardless of the airway device used. This may explain the higher values of systemic arterial blood pressure observed in our study.

A limitation of this study may be represented by the use of human based laryngeal masks rather than the supraglottic airway devices licenced specifically for use in rabbits. Despite the fact that the veterinary version of these devices has been produced for rabbits, to date no study found a clear advantage or disadvantage over the human devices [12]. Rabbits were breathing spontaneously and, in this condition, both devices have shown to deliver the volatile agent adequately although a leak test was not performed. Moreover, tongue cyanosis or other side effects as gastric tympanism or haemoglobin oxygen saturation lower than 95% were not reported with the laryngeal mask [11, 12]. The occurrence of partial occlusion of the larynx by the device may not be excluded without imaging techniques; effects on intraoperative respiratory variables were clinically irrelevant in case of occurrence. The use of size 1 human designed laryngeal mask was adequate for the size of the rabbits enrolled, although for smaller animals the device would have failed. Lastly, all data were obtained retrospectively and may have suffered from bias associated with missing information or nonuniform record keeping. Moreover, this is a single-centre study with a single operator performing all the anaesthetic procedures; although this may limit the variability in the observations, it may represent a bias as volatile agent settings were not standardized but based on the anaesthetist’s discretion.

## Conclusion

The use of a laryngeal mask or an endotracheal tube did not affect the post-induction respiratory variables and it showed no sparing effect on intraoperative volatile agent consumption. Although the dose of isoflurane was not different between groups, rabbits with the laryngeal masks applied were extubated earlier. The combination of dexmedetomidine, midazolam and buprenorphine allowed the insertion of a laryngeal mask device in most of the rabbits without additional propofol, and those agents may have contributed to attenuate the variability on the cardiovascular and respiratory variables. Due to the retrospective nature of this study, further research is warranted to confirm our results.

## Data Availability

The datasets used and/or analysed during the current study are available from the corresponding author on reasonable request.
